# Parafoveal pre-processing in children reading English: The importance of external letters

**DOI:** 10.3758/s13423-020-01806-8

**Published:** 2020-09-11

**Authors:** Sara V. Milledge, Hazel I. Blythe, Simon P. Liversedge

**Affiliations:** 1 University of Southampton, Building 44, Highfield Campus, SO17 1BJ UK; 2 Northumbria University, Newcastle upon Tyne, UK; 3 University of Central Lancashire, Preston, UK

**Keywords:** Reading, Parafoveal pre-processing, Children, English, Internal letters, External letters

## Abstract

Although previous research has demonstrated that for adults external letters of words are more important than internal letters for lexical processing during reading, no comparable research has been conducted with children. This experiment explored, using the boundary paradigm during silent sentence reading, whether parafoveal pre-processing in English is more affected by the manipulation of external letters or internal letters, and whether this differs between skilled adult and beginner child readers. Six previews were generated: identity (e.g., *monkey*); external letter manipulations where either the beginning three letters of the word were substituted (e.g., *rackey*) or the last three letters of the word were substituted (e.g., *monhig*); internal letter manipulations; e.g., *machey*, *mochiy*); and an unrelated control condition (e.g., *rachig*). Results indicate that both adults and children undertook pre-processing of words in their entirety in the parafovea, and that the manipulation of external letters in preview was more harmful to participants’ parafoveal pre-processing than internal letters. The data also suggest developmental change in the time course of pre-processing, with children’s pre-processing delayed compared to that of adults. These results not only provide further evidence for the importance of external letters to parafoveal processing and lexical identification for adults, but also demonstrate that such findings can be extended to children.

## Introduction

In recent years a number of studies have been reported that examine eye-movement behaviour during silent sentence reading in children compared to adults (see Blythe & Joseph, 2011, and Blythe, 2014, for reviews); however, this research has predominantly focused on foveal reading processes. That is, examining word-identification processes for the directly fixated word (*n*). In contrast, there is a paucity of research that directly compares parafoveal reading processes in adults and children, examining how identification of the upcoming word (*n*+1) occurs and which factors can affect such processing.

**Fig. 1:**
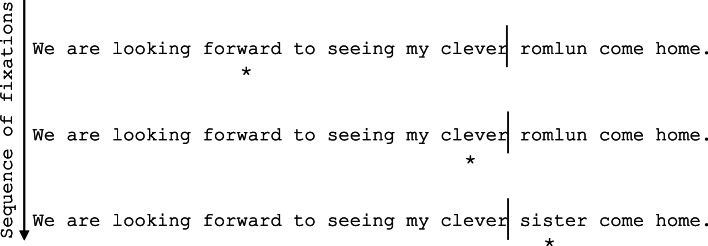
Example of the boundary paradigm (Rayner, 1975). Fixation locations are marked by the asterisk under the sentence. When a sentence is first presented on the screen, the target word is replaced with a preview letter string. When the participant is fixating the pre-target word (*n*-1; *clever* in this example), word *n* (e.g., *sister*) is unavailable for pre-processing. An invisible boundary is placed immediately in front of the target word (marked here by a vertical line for demonstration, though this is not visible on the participant's screen during the experiment). When the reader makes a saccade across the invisible boundary, the preview letter string (e.g., *romlun*) is replaced with the correctly spelled word and the reader is typically unaware that any change has occurred. Two control conditions are typically included – an identity condition, where the preview is identical to the target word, and a completely unrelated preview condition, where all letters are replaced with stimulus strings that do not provide any useful information about the upcoming word (e.g., *romlun*, as shown here). Reading times are typically shortest in the identity condition, as the reader has benefitted from undisrupted parafoveal pre-processing of the target word. Conversely, reading times are expected to be longest in the unrelated preview condition, as the reader has been unable to extract any information that might facilitate lexical identification. Experimental conditions then manipulate/preserve features of the upcoming word as per the manipulations of interest in the study. Reduced reading times on a target word observed after a correct (identity) preview, compared to an incorrect preview (i.e., the experimental conditions and the unrelated preview condition), is known as preview benefit

The use of eye-movement recordings in order to study reading is a dominant research method for skilled adults, providing a moment-to-moment index of the reader’s cognitive processing of text (e.g., Rayner, 2009). Critically, such research has shown that, during a fixation on *n*, adults both process *n* and also begin to pre-process *n*+1. Subsequently, when *n*+1 is directly fixated, reading times are faster due to the pre-processing that has already occurred (see Schotter, Angele, & Rayner, 2012, for a review). This is referred to as parafoveal pre-processing, and can be considered a hallmark of skilled, fluent adult reading (Rayner, Liversedge, & White, 2006a). The importance of parafoveal pre-processing has been shown through a number of studies that have used gaze-contingent paradigms, where the stimulus changes as the reader progresses through the sentence dependent on the location of their fixation (e.g., the boundary paradigm; Rayner, 1975; see Fig. 1). Specifically, gaze-contingent techniques can be used to deny readers the opportunity for parafoveal pre-processing. It is quite clear that skilled adult readers depend upon parafoveal pre-processing for rapid, fluent sentence reading.

In order to gain insight into how beginner readers progress to be skilled readers, it is crucial to understand how this skill, so pivotal to skilled adult reading, develops. Through the boundary paradigm, by manipulating certain characteristics of the relationship between the preview letter string and the correct target word, it is possible to determine the type of information that is pre-processed in the parafovea. Adults pre-process orthography (a word’s printed form), for example displaying faster reading times after an orthographically similar preview is available compared to an orthographically dissimilar preview (e.g., *cahc* vs. *picz* as preview for *cake*; Balota, Pollatsek, & Rayner, 1985). The external letters of a word are particularly important for skilled adult readers in both parafoveal pre-processing (Johnson, Perea, & Rayner, 2007) and during subsequent direct fixation (Johnson & Eisler, 2012). Manipulations that affect the first or final letter of a word have a disproportionately large cost to reading times, relative to manipulations of internal letters, with the first letter seeming to play a particularly important role (e.g., Briihl & Inhoff, 1995; Inhoff, 1989a,b; White, Johnson, Liversedge, & Rayner, 2008).

Little research, however, investigating children’s parafoveal pre-processing in alphabetic languages has been undertaken.^1^ One study has examined the first-letter advantage in parafoveal preview for children compared to adults. Pagán, Blythe, and Liversedge (2016) examined 8- to 9-year-old English children's orthographic pre-processing of the first three letters of an upcoming word. Similar to adults in terms of both the magnitude and the time course of their pre-processing, they also found that children showed a beginning bigram (the first two letters of a word) bias. This study only manipulated the first three letters of words in parafoveal preview, though, and orthographic pre-processing of the entire word form was not examined. Johnson, Oehrlein, and Roche (2018) have also provided evidence for the importance of first letters to children’s pre-processing: faster reading times were found when the first two letters of target words were maintained in preview – orthographically similar condition, compared to when all letters were substituted in preview – orthographically dissimilar condition (e.g., *apydo* vs. *egydo* as previews for *apple*). Thus, the beginning letters clearly play an important role in both adults’ and children’s parafoveal pre-processing, but, whilst Johnson et al.’s (2018) study might suggest that children extract orthography from the entire word in preview, whether children show external letter advantages for both first and final letters or whether this bias is limited to the first letters of a word is unknown.

In the present study two key questions were addressed: (1) whether children are able to pre-process whole target words in the parafovea; and (2) whether external or internal letters are more facilitative to parafoveal pre-processing. To examine these questions the boundary paradigm (Rayner, 1975) was used. The locations of letter substitutions within a target word were manipulated in preview to examine the spatial extent of orthographic pre-processing in children compared to adults – letters were substituted in preview at the beginning, middle, or end of the target words. Research using other experimental paradigms has indicated that children do pre-process some information up to 11 character spaces away from the point of fixation, although those studies did not show which lexical characteristics were processed (e.g., word length, word shape, letter identity, etc.; Häikiö, Bertram, Hyönä, & Niemi, 2009; Rayner, 1986; Sperlich, Schad, & Laubrock, 2015). On this basis, we predicted that both adults and children would be sensitive to letter substitutions at the end of the target word as well as at the beginning. We also expected to show a higher cost to both adults' and children's reading from manipulations that involved the first letters of a word compared to those that involved internal letters within a word (Pagán et al., 2016; White et al., 2008).

## Method

### Participants

Forty-two adults (*M*_*age*_ = 22.24 years) and 42 children (aged 8–9 years; *M*_*age*_ = 8.76 years) participated in the eye-tracking experiment. See Table 1 for a summary of group characteristics. All had normal or corrected-to-normal vision, and were native speakers of English with no known reading difficulties. This was confirmed by the reading subtests of the Wechsler Individual Achievement Test II UK (WIAT-II UK; Wechsler, 2005); all participants were within the expected range (adults’ composite standardised score range: 99–135; children’s composite standardised score range: 104–123; see also Table 1).

**Table 1:** Summary of group characteristics

		Mean	StDev	*t*	*df*	*p*
Test age (years)	Adults	22.24	3.54			
	Children	8.76	.43			
WIAT word reading	Adults	112.17	4.94			
	Children	111.48	4.40	.68	82	.501
WIAT pseudoword decoding	Adults	111.07	5.84			
	Children	109.67	4.80	1.21	82	.232
WIAT comprehension	Adults	119.52	4.65			
	Children	110.21	5.59	8.30	82	< .001
WIAT composite standardised scores	Adults	122.26	8.13			
	Children	110.95	4.77	7.78	82	< .001

*Note.* The three right-hand columns give the results of independent samples *t*-tests comparing the adults to the children. The WIAT scores all refer to standardised scores

### Materials and design

We used the stimuli developed by Pagán et al. (2016), which consisted of 26 target words in sentence frames. These were supplemented by 34 additional target words and sentence frames that we created. Target words were either nouns or adjectives, and were bisyllabic with a CVCCVC structure, with the syllable boundary falling between the second and third consonants (see Table 2 for target-word properties). All materials were pre-screened for both the difficulty of the sentences and the predictability of the target words within each sentence, to confirm that the materials were suitable for use with our target age range. For the additional 34 target words, two possible sentence frames were created. Eighty children (8- to 9-year-olds; none of whom took part in the eye-tracking experiment) rated these sentences on a scale of 1 (easy to understand) to 7 (difficult to understand). They also completed a sentence-constraint rating (predictability) task for the 94 sentences (as Pagán et al., 2016, did not pre-screen for predictability), where the sentence frame was presented with a blank space in the target location and the children were asked to fill in the word that they thought best completed the sentence. The results from the pre-screening are shown in Table 2, and the final stimulus set was selected to ensure that the sentences were easy to understand for our target age range, and that the target word in each sentence was not highly predictable (to minimise skipping). For each of the new target words, one sentence frame was selected for use in the eye-movement experiment on the basis of this pre-screening. Six target words and their associated sentence frames were dropped (one from Pagán et al., 2016). The final stimulus set consisted of 54 experimental sentences.

**Table 2:** Linguistic properties of the target words and sentence frames

	Target words
Orthographic neighbours (N-Watch; Davis, 2005)	≤ 7
Age of Acquisition (Kuperman, Stadthagen-Gonzalez, & Brysbaert, 2012)	*M* = 5.81 years *SD* = 1.63
Child frequency counts (Children’s Printed Word Database; Masterson, Dixon, Stuart, Lovejoy, & Lovejoy, 2003)	Range = 3–663 per million *M* = 85 *SD* = 128
Adult frequency counts (English Lexicon Project Database; HAL corpus, Balota et al., 2007)	Range = 0–2,160 per million *M* = 134 *SD* = 324
Understandability (1 *easy* to 7 *difficult*)	Range = 1–1.63 *M* = 1.14
Predictability	Range = .05–.86 *M* = .34

*Note.* The Ages of Acquisition refer to 50 of the target words, as this information was not available in the database for four of the target words (*conker*, *longer*, *ledges*, and *fences*)

The boundary paradigm (Rayner, 1975) was used. Using this paradigm, the text displayed on the screen changes contingent on where the reader is fixating (see Fig. 1). A preview letter string occupies the target word location at trial onset, but when the reader makes a saccade to directly fixate the target word (crossing an invisible boundary), the preview letter string changes to the correct target word. In the current experiment, six parafoveal preview conditions (or letter strings) were generated for each target word (see Appendix 1). There were two control conditions: an identity condition, where the preview was identical to the target word (123456; e.g., *sister* – *sister*), and an unrelated condition, where only the letter shapes of the target word were maintained in preview (dddddd; e.g., *romlun* – *sister*). There were four other experimental conditions which each involved the substitution of three of the letters of the target words in preview: the beginning three letters of each word (ddd456); internal letters 2, 3, and 4 (1ddd56); internal letters 3, 4, and 5 (12ddd6); and the end three letters of each word (123ddd). Both the beginning and end substitution conditions were within one syllable, whilst the middle substitution conditions affected both syllables. Both CVCCVC structure and word shape were maintained in these substitutions.

The 54 experimental sentences were counterbalanced across six lists using a Latin-square design (nine sentences per condition). The sentences occupied one line on the screen (maximum = 77 characters; *M* = 60 characters) and each target word was placed near the middle of the sentence.

### Apparatus and procedure

An EyeLink 1000 eye-tracker recorded right eye movements (SR Research). Forehead and chin rests were used to minimise head movements. The sentences were presented in 14-pt black Courier New font on the grey background of a 21-in. CRT monitor, with a refresh rate of 120 Hz, at a 60-cm viewing distance; one character subtended .34° of visual angle. Participants were instructed to read normally and for comprehension. Once participants had finished reading a sentence, they pressed a response key, and one-third of the sentences were replaced by a comprehension question, to which the participants responded. After completion of the experiment, participants were asked whether they had noticed anything strange about the appearance of the sentences in the experiment: detecting a display change can affect fixation times (e.g., White, Rayner, & Liversedge, 2005). Four adult participants reported noticing something unusual about the sentences, so their data were excluded from the analyses. The whole experiment lasted about 45 min per participant.

## Results

All participants scored at least 78% correct on the comprehension questions (adults: *M* = 98%; children: *M* = 92%). The data were trimmed using the clean function in DataViewer (SR Research).^2^ In total 1,886 fixations were merged or deleted (2.36% of the dataset; 693 adult fixations, and 1,193 child fixations).

Reading-time data on the target word in each sentence were analysed. Before analysing the local dependent measures, the data were further cleaned: trials in which the boundary change occurred early during a fixation on the pre-target word, and those that occurred late when the display change was not completed until more than 15 ms after onset of fixation on the target word were excluded from the analyses (230 adult trials – 10.14% of the adult trials, and 314 children’s trials – 13.84% of the children’s trials).^3^ Prior to analysis, reading-time data were log transformed.

Data were analysed using linear mixed effects (lme) models, using the lmer function from the lme4 package (Bates, Mächler, Bolker, & Walker, 2015) within the R environment for Statistical Computing (R Core Team, 2020). We focus here upon three dependent measures: first fixation duration (the duration of the initial first-pass fixation on a word, regardless of how many fixations the word received), gaze duration (the sum of all fixations on the word before the eyes left it for the first time), and total reading time (the sum of all fixations made on the target word) (see Table 3). Participants and items were entered as crossed random effects. A full random structure was initially specified for participants and items, to avoid being anti-conservative (Barr, Levy, Scheepers, & Tily, 2013); the random structure was trimmed until the models converged. Effects were considered significant when, initially, |*t*| > +/-1.96.

**Table 3:** Mean and standard deviation (in parentheses) reading times on the target word in each condition

Group	Condition	First-fixation duration (ms)	Gaze duration (ms)	Total reading time (ms)
Adults	123456	220 (66)	245 (84)	330 (186)
	ddd456	255 (89)	293 (109)	415 (234)
	1ddd56	254 (90)	291 (121)	395 (243)
	12ddd6	246 (78)	285 (99)	390 (224)
	123ddd	265 (98)	304 (118)	424 (275)
	dddddd	259 (79)	310 (131)	427 (241)
Children	123456	290 (141)	505 (508)	726 (671)
	ddd456	320 (159)	529 (367)	790 (591)
	1ddd56	293 (130)	505 (343)	773 (612)
	12ddd6	294 (144)	515 (387)	733 (511)
	123ddd	298 (162)	580 (594)	851 (745)
	dddddd	309 (154)	529 (475)	775 (594)

In all of the lme models there were significant group differences: children displayed significantly longer first fixations, gaze durations, and total reading times than the adults (see Table 3). We focus upon significant effects of the experimental manipulations, and any interactions with participant group.^4^

### Model 1

This model used the identity control condition (123456) as a baseline, with each of the non-word preview conditions compared to it, thus examining the potential costs associated with substitutions being present in the parafovea, and the extent to which participants were gaining preview benefit. As can be seen from Tables 3 and 4, for all of the non-word preview conditions the adults experienced a significant cost relative to the identity condition – their foveal word identification was facilitated by obtaining a processing benefit from the correct parafoveal preview. The presence of significant interactions with participant group suggests that adults and children differed in their processing of letter substitutions in preview, in the earlier measure of first fixation duration. In contrast to the adults, children showed little increase in reading times for any of the substitution conditions, with the exception of ddd456, demonstrating a lack of preview benefit. Clearly, both adults and children, though, experienced a cost to early measures of lexical processing when parafoveal pre-processing of the first letter of the word was disrupted. Substitutions of other letters in the word disrupted very early lexical processing for adults but not children, who showed delayed sensitivity to substitutions of all except the first letter of the word. Certainly, by the time the reader had engaged in second-pass reading on a word, both adults and children showed a cost to reading times from substitutions in all letter positions in preview, demonstrating comparable preview benefit effects.^5^

**Table 4:** Output from Model 1 for first-fixation duration, gaze duration and total reading time

	First-fixation duration				Gaze duration				Total reading time			
	b	SE	*t*	*p*	b	SE	*t*	*p*	b	SE	*t*	*p*
Adults, 123456 (Int)	5.35	.03	184.63	< .001	5.44	.05	113.14	< .001	5.67	.06	101.81	< .001
**Adults, Children**	**.22**	**.04**	**5.34**	**< .001**	**.52**	**.06**	**8.04**	**< .001**	**.65**	**.07**	**8.84**	**< .001**
**Adults, ddd456**	**.13**	**.03**	**4.72**	**< .001**	**.17**	**.03**	**5.27**	**< .001**	**.23**	**.04**	**6.20**	**< .001**
**Adults, 1ddd56**	**.13**	**.03**	**4.73**	**< .001**	**.16**	**.03**	**4.93**	**< .001**	**.18**	**.04**	**4.76**	**< .001**
**Adults, 12ddd6**	**.10**	**.03**	**3.59**	**< .001**	**.15**	**.03**	**4.61**	**< .001**	**.17**	**.04**	**4.57**	**< .001**
**Adults, 123ddd**	**.17**	**.03**	**6.02**	**< .001**	**.21**	**.03**	**6.47**	**< .001**	**.23**	**.04**	**6.03**	**< .001**
**Adults, dddddd**	**.16**	**.03**	**5.69**	**< .001**	**.23**	**.03**	**6.94**	**< .001**	**.26**	**.04**	**6.95**	**< .001**
Children × ddd456	-.05	.04	-1.23	.220	-.06	.05	-1.25	.211	-.08	.05	-1.55	.121
**Children × 1ddd56**	**-.12**	**.04**	**-2.90**	**.004**	-.08	.05	-1.61	.108	-.05	.05	-.97	.331
**Children × 12ddd6**	**-.09**	**.04**	**-2.37**	.018*	-.07	.05	-1.44	.151	-.07	.05	-1.38	.167
**Children × 123ddd**	**-.16**	**.04**	**-3.91**	**< .001**	-.09	.05	-1.80	.072	-.05	.05	-1.02	.309
**Children × dddddd**	**-.11**	**.04**	**-2.65**	.008*	**-.15**	**.05**	**-3.27**	**.001**	**.13**	**.05**	**-2.51**	.012*

*Note.* The reading-time data were log transformed prior to analysis, so the model estimates cannot be directly interpreted. Significant effects are indicated in bold. The syntax, following trimming, for first-fixation duration, gaze duration, and total reading time as intercepts only models was as follows: depvar ~ Group * condition + (1|Participant) + (1|targetno). The *s denote where significance levels changed with the use of the *glht* function (i.e., where results went from being significant to non-significant/marginally significant- within first fixation duration: *p* = .130 and *p* = .065, respectively, and within total reading time: *p* = .093)

### Model 2

**Fig. 2:**
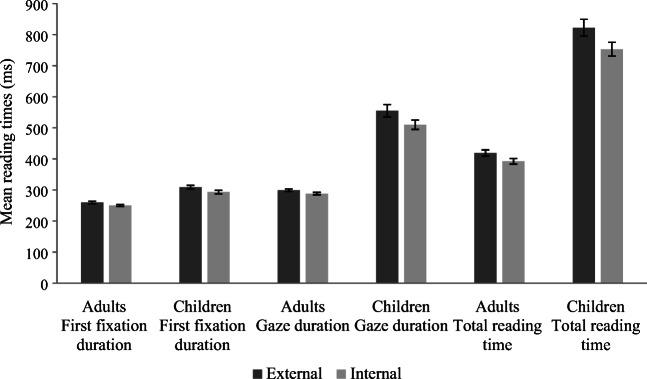
Mean reading times for the collapsed external letter substitution conditions (ddd456 and 123ddd) and the internal letter substitution conditions (1ddd56 and 12ddd6), for both adults and children

This model collapsed ddd456 and 123ddd together, and 1ddd56 and 12ddd6 together, in order to compare external to internal letter manipulations. The *contr.sdif* function (package MASS) was used to set up the factors. Then, contrasts were run to compare ddd456 to 123ddd for adults and children separately. As shown in Table 5, and Fig. 2, the internal letter substitution conditions led to significantly faster reading times than the external letter substitution conditions, for both adults and children. Also, the contrasts revealed that, in first fixation duration, the children were showing a first-letter bias. Children’s reading times were significantly slower in ddd456 than 123ddd in this very early measure of processing (see Table 3). Interestingly, note that in gaze duration and total reading time this effect of external letter substitutions seemed mainly to be driven by the end letter (123ddd; see Table 3).

**Table 5:** Output from Model 2, and contrasts, for first-fixation duration, gaze duration, and total reading time

	First-fixation duration				Gaze duration				Total reading time			
	b	SE	*T*	*p*	b	SE	*t*	*p*	b	SE	*t*	*p*
Intercept	5.54	.02	322.03	< .001	5.84	.03	183.00	< .001	6.16	.04	162.44	< .001
**Adults, Children**	**.11**	**.03**	**3.39**	**.001**	**.45**	**.06**	**8.07**	**< .001**	**.59**	**.07**	**8.99**	**< .001**
**External vs. Internal**	**-.03**	**.01**	**-2.44**	**.015**	**-.04**	**.02**	**-2.25**	.024*	**-.05**	**.02**	**-2.92**	**.004**
Group × External vs. Internal	-.0003	.03	-.01	.991	.001	.03	.03	.978	.005	.04	.13	.894
*Contrasts*												
Intercept	5.53	.02	314.32	< .001	5.82	.04	144.40	< .001	6.14	.05	124.05	< .001
Adults, ddd456 - 123ddd	-.04	.03	-1.31	.189	-.04	.03	-1.22	.223	.006	.04	.17	.868
**Children, ddd456 - 123ddd**	**.07**	**.03**	**2.34**	**.019**	-.02	.03	-.57	.568	-.03	.04	-.74	.461

*Note.* The reading-time data were log transformed prior to analysis, so the model estimates cannot be directly interpreted. Significant effects are indicated in bold. The syntax for first-fixation duration, gaze duration, and total reading time following trimming, as intercepts only models, was as follows: depvar ~ Group * CollCons + (1|Participant) + (1 | targetno). The contrasts were set up for first-fixation duration, gaze duration, and total reading time within the following syntax (intercepts only models following trimming): *depvar ~ GroupByCond + (1 | Participant) + (1 | targetno)*. In order to use the *glht* function for Model 2, contrasts were set up for all dependent measures within the following syntax: depvar ~ Group * condition3 + (1|Participant) + (1|targetno). The * denotes where the significance level changed with the use of the *glht* function (i.e., where the result went from being significant to marginally significant- *p* = .071)

### Controlling for multiple comparisons

Given that Models 1 and 2 contain a number of comparisons across the five experimental conditions, we ran these models again using the *glht* function (package multcomp) to adjust *p* values and control for the multiple comparisons being made within each model (Hothorn, Bretz, & Westfall, 2008).^6^ For the majority of effects, this did not change the pattern of significance; we report here those instances where the correction did make a difference. First, within first fixation duration in Model 1, the interaction term between children and 12ddd6 became non-significant (and marginally significant between children and dddddd), suggesting that the children’s parafoveal pre-processing in these conditions was not significantly different (or only marginally so) to that of the adults.^7^ Second, the interaction between children and dddddd became non-significant in total reading time; here, the children’s processing was consistent with that of the adults (see also Table B2). Third, within Model 2, in gaze duration, the main effect of external compared to internal letter substitutions in preview became marginally significant.

## Discussion

The present study investigated parafoveal pre-processing in English children and adults during silent sentence reading, specifically comparing pre-processing of beginning, internal and end letters. As expected, the children did pre-process the whole target word in the parafovea. Like adults, they displayed a cost from 123ddd substitutions, demonstrating that they were sensitive to substitutions of the final letter of the target words (albeit a slightly delayed effect, i.e., present in gaze duration). This indicates that children’s parafoveal pre-processing (of *n*+1) was not constrained by visual acuity limitations. If pre-processing was constrained by visual acuity, 123ddd should have been the least disruptive condition, as those substitutions were furthest away from the point of fixation. Instead, the significant cost associated with end-letter substitutions clearly demonstrates that children's parafoveal pre-processing extended over the orthographic form of the whole word (six letters, in this case), rather than being constrained to the first few letters.

The data are suggestive of children’s processing being delayed compared to the skilled adult readers, with a developmental change in the time course of pre-processing: adults showed early effects in first fixation duration, whilst the two groups only patterned similarly in later processing. This is consistent with children's rate of lexical processing being slower than that of adults, as found by the E-Z Reader model when used to simulate adults’ and children’s eye movement behaviour during reading (Reichle et al., 2013). If children are slower to process word *n* then it stands to reason that they will also be slower to pre-process information from *n*+1. Consequently, each word in the sentence is pre-processed to a reduced degree, and is processed at a slower rate during direct fixation for a child compared to an adult. It is, therefore, unsurprising that children's overall reading times on words were longer, and that effects were delayed in children compared to adults.

This study provides strong evidence for the importance of external letters in children's lexical identification, consistent with skilled adult readers. As shown by collapsing, respectively, the internal and the external letter substitutions together, both adults and children benefitted from faster reading times when the internal letters (1ddd56 and 12ddd6), relative to the external letters (ddd456 and 123ddd), were substituted. Thus, consistent with the literature on skilled adult reading (White et al., 2008), the identity of a word's external letters facilitated children's parafoveal pre-processing more than its internal letters. With respect to syllabic boundaries, the conditions that substituted letters in both syllables of a word (1ddd56 and 12ddd6) were less disruptive to pre-processing than conditions that substituted letters in just one syllable (ddd456 and 123ddd). Thus, external letters are critical to parafoveal pre-processing, to a far greater degree than any pre-processing of syllabic structure.

These results are consistent with Grainger and Ziegler’s (2011) model of orthographic processing. Both the adults and the children, albeit delayed, appeared to be using coarse-grained orthographic processing.^8^ The benefits gained from the internal letter substitutions, relative to the external letter substitutions, suggest that both groups were not sensitive to the absolute precise ordering of letters in preview, but were rather coding for the most visible letters that best constrained word identity and facilitated lexical identification – the external letters. This is broadly supportive of flexible letter position encoding models (e.g., SOLAR, Davis, 2010; SERIOL, Whitney, 2001).

The delay in the children’s pre-processing of orthography (preview benefit) compared to the adults could be due to orthographic representations being less precisely encoded in the children (e.g., Perfetti, 2007). When letter substitutions were present in preview this came at an immediate cost to the adults compared to the identity condition, whilst this effect was delayed in the children. If orthographic forms are less precisely encoded in children, they would experience less of an immediate cost when orthography is manipulated in preview, in contrast to the adults with their more precisely encoded orthographic representations, who would be more reliant on the presence of whole-word orthography in preview (as provided by the identity condition). Consequently, there would appear to be a developmental change in the tuning of orthographic word-recognition processes (e.g., Castles, Davis, Cavalot, & Forster, 2007).

One unexpected result was the lack of a first-letter bias in the adults, that is a more important role in preview for the first letter than the final letter, as found in previous studies (e.g., White et al., 2008), though when first and final letters were collapsed into a single, “external” condition, this was significantly different to internal letter substitutions (consistent with previous research). The present study did ultimately find though that the first letter of the target words was important to adults’ pre-processing (albeit not more so than the final letter); substituting the first letters in preview (ddd456) came at a significant cost relative to the identity condition. It may be that the finding of a first-letter bias depends on the exact nature of the experimental manipulation. Most research has looked at letter transpositions, not substitutions (e.g., Johnson & Eisler, 2012; Rayner, White, Johnson, & Liversedge, 2006b; White et al., 2008). Importantly, though, Johnson et al. (Experiment 3; 2007), showed that both first-letter transposition and substitution previews were detrimental to reading times.^9^ Consequently, we would have expected an effect of first-letter substitutions in the adults. The lack of this effect could be due to the stimuli which, here, were specifically designed for children and would, therefore, have been very easy for the skilled adult readers. The adults’ ease of processing for these sentences may have resulted in a greater degree of parafoveal pre-processing for the target word than would be the case with more difficult sentences (e.g., Henderson & Ferreira, 1990). Thus, the adult readers may have allocated their attention across the entire form of *n*+1 (not just the initial letters). For the adults, consequently, both the first and final (external) letters were important to their pre-processing.

Children, similar to the adults, displayed sensitivity to first-letter substitutions very early in their lexical processing – in first-fixation duration. The 30-ms preview benefit effect found within this measure in the children was comparable in size to the effect found within the adults (35 ms). This suggests that the privileged status of the first letter/s to lexical identification is evident very early in both adults’ and children’s lexical processing, especially given how this information was manipulated parafoveally. Whilst the adults, though, did not show a first-letter bias (comparing ddd456 against 123ddd), the children did. This evidence for the importance of the first letter in children’s pre-processing is consistent with Pagán et al. (2016) and Johnson et al. (2018), who found numerical trends for a bias towards the first bigram of target words in all dependent measures for children.^10^ Overall, the evidence strongly suggests that the first letter/s of words are important for facilitating children’s lexical identification in preview.

There are several reasons why the first letter of a word might be particularly important for lexical identification. One possibility is reduced lateral masking, or crowding, due to the inter-word space on one side, whilst internal letters are subject to greater lateral masking from the presence of other letters on both sides (e.g., Bouma, 1973; Levi, 2008). Alternatively, it could be more cognitively based, in that identification of the first letter of a word could drive the process of lexical identification. Certainly, Johnson and Eisler’s (2012) research, with adults, suggests this could be the case. For example, they found that when lateral masking was equated by replacing inter-word spaces with #s (e.g., The#boy#could#not#solve#the#problem#so#he#asked#for#help.), first letter transpositions were still significantly more difficult for readers than internal transpositions, whilst final letter transpositions were no more harmful than the internal transpositions (Experiments 1 and 2). This suggests a critically important role for the first letter of a word in lexical identification, irrespective of low-level visual factors like crowding. This finding contrasts with effects associated with a word’s final letter.

In summary, the present study provides novel evidence of children pre-processing whole words during English reading, and experiencing costs from external letter manipulations in preview, similar to adults. External letters appear to play a specific and important role in visual word recognition, seeming to fundamentally relate to how both adult and child readers access lexical information.

## Data Availability

The data that support the findings of this study, and the code used for the main model analyses, are available from: https://osf.io/gbsmf/?view_only=41de4a5d7dca4c058690aca15cafc8a6. The experiment was not preregistered.

## Appendix 1

Experimental sentences and preview conditions (ddd456, 1ddd56, 12ddd6, 123ddd, and dddddd):

The blonde girl spotted the brown *monkey* in the zoo. (*rackey*, *machey*, *mochiy*, *monhig*, *rachig*)

Tom got an appointment with the nice *doctor* in the hospital. (*bintor*, *dinfor*, *donfur*, *docfur*, *binfur*)

Peter put clothes in the laundry *basket* ready for washing. (*hurket*, *burlet*, *barlit*, *baslik*, *hurlik*)

You can find nice fruit in the local *market* on Tuesdays. (*wonket*, *mondet*, *mandit*, *mardil*, *wondil*)

Kelly always chooses her lucky *number* to play the lottery. (*savber*, *navter*, *nuvtor*, *numtoc*, *savtoc*)

The man was in grave *danger* as he climbed the mountain. (*homger*, *domper*, *dampir*, *danpis*, *hompis*)

We saw a large *badger* when we went for a walk last night. (*hilger*, *bilper*, *balpur*, *badpun*, *hilpun*)

We did not stay much *longer* than you at the birthday party. (*tumger*, *lumjer*, *lomjar*, *lonjaw*, *tumjaw*)

I like the grey *donkey* that lives in a field behind my house. (*farkey*, *dartey*, *dortiy*, *dontip*, *fartip*)

Daniel drew a picture with a green *pencil* for his grandma. (*jumcil*, *pumril*, *pemral*, *penrab*, *jumrab*)

The letter was stuck with a large *magnet* on our fridge door. (*voynet*, *moyret*, *mayrut*, *magrud*, *voyrud*)

My uncle has a short *temper* and shouts when I’m naughty. (*dowper*, *towger*, *tewgar*, *temgan*, *dowgan*)

Sue got her hair cut shorter than *normal* and it looked nice. (*cusmal*, *nusval*, *nosvil*, *norvib*, *cusvib*)

The baby fell asleep after many *tender* kisses from his mum. (*basder*, *tasfer*, *tesfir*, *tenfim*, *basfim*)

I put lots of silver *tinsel* on the Christmas tree this year. (*famsel*, *tamrel*, *timrul*, *tinrud*, *famrud*)

The oil was stored in a huge *tanker* until it was needed. (*lucker*, *tacder*, *tacdor*, *tandos*, *lucdos*)

My football team’s *mascot* is a giant teddy bear in uniform. (*vixcot*, *mixrot*, *maxret*, *masrel*, *vixrel*)

The little boy is a real *rascal* because he plays jokes on people. (*wencal*, *renmal*, *ranmul*, *rasmut*, *wenmut*)

My neighbours planted a small *conker* tree in their garden. (*simker*, *cimber*, *combur*, *conbux*, *simbux*)

The new building has window *ledges* that are painted blue. (*hubges*, *lubpes*, *lebpas*, *ledpar*, *hubpar*)

Tom cried when his little *finger* got caught in the door. (*tasger*, *fasyer*, *fisyur*, *finyum*, *tasyum*)

The horse jumped six white *fences* and won the competition. (*larces*, *farmes*, *fermis*, *fenmix*, *larmix*)

The ambulance took the hurt *victim* quickly to the hospital. (*surtim*, *vurlim*, *virlom*, *viclon*, *surlon*)

The front *bumper* fell off dad’s car today and he was cross. (*hinper*, *binjer*, *bunjar*, *bumjas*, *hinjas*)

The boss bought a new *dumper* truck for the building project. (*ticper*, *dicyer*, *ducyar*, *dumyas*, *ticyas*)

The castle has a large *garden* which we like to play in. (*pocden*, *gochen*, *gachun*, *garhum*, *pochum*)

The space museum had a new model *rocket* ride that was brilliant. (*wasket*, *rasbet*, *rosbit*, *rocbil*, *wasbil*)

The couple decided to buy a cream *carpet* to go in the bedroom. (*nimpet*, *cimget*, *camgut*, *cargud*, *nimgud*)

My aunt’s chatty *parrot* learns new words very quickly and is very clever. (*jesrot*, *pescot*, *pascut*, *parcuf*, *jescuf*)

Bob looked down out of the attic *window* to the street below. (*rasdow*, *waslow*, *wisluw*, *winlum*, *raslum*)

I had some really tasty *turkey* in my sandwich today. (*dimkey*, *timley*, *tumlay*, *turlag*, *dimlag*)

The children were excited about the great *circus* that was coming to town. (*mancus*, *canxus*, *cinxes*, *cirxen*, *manxen*)

The photo was of a field with a tiny *piglet* playing in it. (*qujlet*, *pujdet*, *pijdat*, *pigdab*, *qujdab*)

Alice saw a very prickly *cactus* during her holiday last summer. (*rintus*, *cinkus*, *cankes*, *cackem*, *rinkem*)

Ben’s parents bought a soft *pillow* for his bed last week. (*gadlow*, *padtow*, *pidtaw*, *piltac*, *gadtac*)

We are looking forward to seeing my clever *sister* come home. (*romter*, *somler*, *simlur*, *sislun*, *romlun*)

Hannah smiled as the happy *butler* let her into the big house. (*hadler*, *badfer*, *budfir*, *butfin*, *hadfin*)

He took the empty *carton* from the fridge and threw it away. (*sixton*, *cixbon*, *caxben*, *carbem*, *sixbem*)

The man ironed his shirt *collar* ready for work the next day. (*mudlar*, *cudtar*, *codter*, *coltes*, *mudtes*)

Kate peeled and cut the juicy *carrot* ready to put in her dinner. (*senrot*, *cenmot*, *canmit*, *carmid*, *senmid*)

The lady put a silky *ribbon* onto the dress she was making. (*makbon*, *raklon*, *riklan*, *riblas*, *maklas*)

The forest was the perfect setting for the family *picnic* last week. (*yawnic*, *pawric*, *piwrac*, *picrum*, *yawrum*)

Following the instructions, Callum mixed the soft *powder* with a cup of water. (*junder*, *punber*, *ponbir*, *powbis*, *junbis*)

Mary crawled down the dirty *tunnel* to try to find her football. (*bacnel*, *tacsel*, *tucsil*, *tunsid*, *bacsid*)

At the animal park there was a huge *walrus* with very long tusks. (*nibrus*, *wibmus*, *wabmes*, *walmen*, *nibmen*)

The children loved to see the kind *puppet* help his friends. (*qagpet*, *pagjet*, *pugjot*, *pupjod*, *qagjod*)

The dress was made of a thin *fabric* that was soft to touch. (*tolric*, *folsic*, *falsuc*, *fabsum*, *tolsum*)

I love to wear my cosy *jumper* for walks when it’s cold outside. (*yawper*, *jawger*, *juwgir*, *jumgis*, *yawgis*)

The man was sent a funny *letter* through the post from his friend. (*hidter*, *lidber*, *ledbar*, *letban*, *hidban*)

The builders decided to put the strong *ladder* up against the wall. (*bufder*, *lufter*, *laftir*, *ladtis*, *buftis*)

Jill was proud of the large *turnip* that she had dug up. (*dacnip*, *tacmip*, *tucmop*, *turmog*, *dacmog*)

I was sent to buy a yellow *pepper* from the supermarket. (*jagper*, *pagqer*, *pegqur*, *pepqum*, *jagqum*)

It was nearly *winter* and I hoped that it would snow. (*comter*, *womder*, *wimdar*, *windas*, *comdas*)

Sam looked up at the stars on the clear *summer* night.

(*nicmer*, *sicver*, *sucvar*, *sumvan*, *nicvan*)

## Appendix 2

Supplementary tables and analyses

**Table 6:** Skipping rates and standard deviations (in parentheses) on the target word in each condition across all participants

Group	Condition	Percentage of skips
Adults	123456	6.85% (.25)
	ddd456	1.78% (.13)
	1ddd56	1.97% (.14)
	12ddd6	2.07% (.14)
	123ddd	1.83% (.13)
	dddddd	1.17% (.11)
Children	123456	7.34% (.26)
	ddd456	5.13% (.22)
	1ddd56	4.92% (.22)
	12ddd6	4.55% (.21)
	123ddd	4.52% (.21)
	dddddd	5.49% (.23)

**Table 7.** Output from Model 1 for first-fixation duration, gaze duration, and total reading time using 123456 as a baseline for adults and children separately

	First-fixation duration				Gaze duration				Total reading time			
	b	SE	*t*	*p*	b	SE	*t*	*p*	b	SE	*t*	*p*
*Adults*												
123456 (Int)	5.35	.02	227.89	< .001	5.45	.03	213.17	< .001	5.67	.04	142.57	< .001
**ddd456**	**.13**	**.02**	**5.70**	**< .001**	**.17**	**.03**	**6.74**	**< .001**	**.23**	**.04**	**6.55**	**< .001**
**1ddd56**	**.13**	**.02**	**5.74**	**< .001**	**.16**	**.03**	**6.39**	**< .001**	**.17**	**.03**	**4.94**	**< .001**
**12ddd6**	**.10**	**.02**	**4.32**	**< .001**	**.15**	**.03**	**5.90**	**< .001**	**.17**	**.04**	**4.75**	**< .001**
**123ddd**	**.17**	**.02**	**7.26**	**< .001**	**.21**	**.03**	**8.31**	**< .001**	**.23**	**.04**	**6.41**	**< .001**
**dddddd**	**.16**	**.02**	**6.88**	**< .001**	**.22**	**.03**	**8.90**	**< .001**	**.26**	**.04**	**7.31**	**< .001**
*Children*												
123456 (Int)	5.57	.03	163.82	< .001	5.97	.06	92.77	< .001	6.32	.07	90.29	< .001
**ddd456**	**.08**	**.03**	**2.45**	**.014***	**.11**	**.04**	**2.77**	**.006**	**.15**	**.04**	**3.75**	**< .001**
**1ddd56**	.02	.03	.47	.639	**.09**	**.04**	**2.14**	**.033***	**.13**	**.04**	**3.26**	**.001**
**12ddd6**	.01	.03	.19	.847	**.08**	**.04**	**2.07**	**.038***	**.10**	**.04**	**2.48**	**.013***
**123ddd**	.01	.03	.39	.696	**-.09**	**.04**	**3.23**	**.001**	**.17**	**.04**	**4.44**	**< .001**
**dddddd**	.06	.03	1.58	.115	-.15	.04	1.82	.069	**.13**	**.04**	**3.33**	**< .001**

*Note.* The reading-time data were log transformed prior to analysis, so the model estimates cannot be directly interpreted. Significant effects are marked in bold. The syntax, following trimming, for first-fixation duration, gaze duration, and total reading time as intercepts only models, for both adults and children, was as follows: *depvar ~ condition + (1|Participant) + (1|targetno)*. The *s denote where the significance levels changed with the use of the *glht* function (i.e., where results went from being significant to non-significant/marginally significant- within first fixation duration: *p* = .059, within gaze duration: *p* = .124 and *p* = .143, respectively, and within total reading time: *p* = .054)

## Footnotes

1 Two studies have been conducted in English (Johnson, Oehrlein, & Roche, 2018; Pagán, Blythe, & Liversedge, 2016) and four in German (Marx, Hawelka, Schuster, & Hutzler, 2015, 2017; Marx, Hutzler, Schuster, & Hawelka, 2016; Tiffin-Richards & Schroeder, 2015).

2 Fixations shorter than 80 ms were merged with the neighbouring fixation if within a .50° distance of another fixation over 80 ms, and fixations shorter than 40 ms were merged with neighbouring fixations if within a 1.25° distance of each other. Then if an interest area had three or more fixations shorter than 140 ms, these were merged into longer fixations. Finally, all remaining fixations shorter than 80 ms or longer than 1,200 ms were deleted.

3 A late boundary change was also operationalised as 10 ms in order to compare the results with the 15-ms report. The pattern of data remained unchanged between the two, so the 15-ms criterion of a late boundary change was used as it allowed the retention of more data (3,992 data points as opposed to 3,837). Regarding the number of items per condition for each participant, after the boundary change cleaning, within the adults the lowest total number of items recorded for a participant was 43 (*M* = 46.52, total range: 42–54; 123456 *M* = 8.00, range: 6–9; ddd456 *M* = 8.02, range: 5–9; 1ddd56 *M* = 8.48, range: 7–9; 12ddd6 *M* = 8.05, range: 5–9; 123ddd *M* = 7.81, range: 4–9; and dddddd *M* = 8.17, range: 6–9) and within the children this was 38 (*M* = 48.52, total range: 38–53; 123456 *M* = 7.79, range: 3–9; ddd456 *M* = 7.43, range: 4–9; 1ddd56 *M* = 7.74, range: 5–9; 12ddd6 *M* = 7.86, range: 5–9; 123ddd *M* = 7.90, range: 6–9; dddddd *M* = 7.81, range: 4–9).

4 In Appendix B, skipping rates are provided in Table B1. No generalized linear mixed models would converge for this measure. In addition, separate analyses were also undertaken for the adults and the children with regard to Model 1, as shown in Table B2.

5 Note that in Table 3, the means suggests that the children were not necessarily patterning in the same way as adults, especially in regard to gaze durations. It is also clear that there was substantially more variability around the means in the children’s data compared to that of the adults. Indeed, within the separate analyses for the children (see Table B2), after controlling for multiple comparisons, the two middle internal letter substitution preview conditions became non-significant; suggesting that, in gaze duration, the external letters being preserved in preview was as facilitative to the children’s lexical identification as the identity condition (whilst the adults experienced significant costs).

6 We did not include the intercept when using the *glht* function, as it was not actively being compared within our models.

7 When examining the children’s first fixation duration results separately though (see Table B2), the children’s processing in these conditions was different to that of the adults: whilst the adults were showing costs in all of the preview conditions compared to the identity preview, the children were not (apart from marginally in the beginning letter substitution preview condition – ddd456).

8 This type of orthographic processing allows direct access from orthography to semantics (meaning). Using this kind of orthographic processing, it is posited that approximate letter positions are coded for within words. This is in contrast to fine-grained orthographic processing, which provides access to semantics via phonological and morphological forms, where sensitivity is shown to the precise order of letters within words.

9 Although no direct comparison was made of the first- and final-letter substitution conditions, differences in condition means suggest that the first-letter substitution condition increased reading times more than the final-letter substitution condition (Table 4, p. 218).

10 It is of note that the analyses undertaken by both Pagán et al. (2016) and Johnson et al. (2018) and the present study are, again, different. Pagán et al.’s study focused on comparing transposed letters to substituted letters (SLs), whilst the present study only examined substituted letters. Also, the present study included a final letter manipulation, Pagán et al.’s study did not. Johnson et al. (2018), similar to the present study, used letter substitutions in preview; however, although a final letter manipulation was present in this study, no direct comparison was, or could be, made with regard to its role in preview in comparison to the first letter, given that the final letter was manipulated in both orthographic preview conditions. Consequently, the closest comparison we could make to that of Pagán et al.’s SL12 versus SL23 effect is a comparison of ddd456 versus 1ddd56. We also show a numerical pattern in our dependent measures between ddd456 and 1ddd56 (first-fixation duration: 27 ms; gaze duration: 24 ms; total reading time: 17 ms); these effects are larger than the largest effect found by Pagán et al. (10 ms in single fixation duration). It is likely that this is due to the different number of letters substituted; whilst Pagán et al. substituted two letters, we substituted three. The size of the effect is almost certain to have increased commensurately with the number of letters substituted. With regard to Johnson et al., the closest comparison we could make to their orthographically dissimilar preview versus their orthographically similar preview effect is dddddd versus 12ddd6. We also show a numerical pattern in our measures between dddddd and 12ddd6 (first fixation duration: 15 ms; gaze duration: 14 ms; total reading time: 42 ms), broadly consistent with their findings for the neutral context, as these results are most applicable to the present research (first fixation duration: 30 ms; gaze duration: 15 ms; total reading time: 19 ms).
